# SNP rs3202538 in 3′UTR region of ErbB3 regulated by miR-204 and miR-211 promote gastric cancer development in Chinese population

**DOI:** 10.1186/s12935-017-0449-z

**Published:** 2017-09-13

**Authors:** Yaxiang Shi, Xuan Chen, Biao Xi, Xiaowen Yu, Jun Ouyang, Chunxia Han, Yucheng Qin, Defeng Wu, Hong Shen

**Affiliations:** 1 Department of Gastroenterology, Zhenjiang Hospital of Traditional Chinese Medicine, Zhenjiang Affiliated Hosptial of Nanjing University of Chinese Medicine, Zhenjiang, China; 2 Department of Gastroenterology, Jiangsu Province Hospital of TCM, Affiliated Hospital of Nanjing University of TCM, Nanjing, China; 3 College of Jingjiang, Jiangsu University, Zhenjiang, China

**Keywords:** ErbB3, MiR-204, miR-211, 3′-UTR, Survival

## Abstract

**Background/aims:**

ErbB3 is an oncogene which has proliferation and metastasis promotion effects by several signaling pathways. However, the individual expression difference regulated by miRNA was almost still unknown. We focused on the miRNAs associated SNPs in the 3′-UTR of ErbB3 to investigate the further relationship of the SNPs with miRNAs among Chinese gastric cancer (GC) patients.

**Methods:**

We performed case–control study including 851 GC patients and 799 cancer-free controls. Genotyping, real-time PCR assay, cell transfection, the dual luciferase reporter assay, western-blot, cell proliferation and trans-well based cell invasion assay were used to investigate the effects of the SNP on ErbB3 expression. Moreover, a 5-years-overall survival and relapse free survival were investigated between different genotypes.

**Results:**

We found that patients suffering from *Helicobacter pylori* (*Hp.*) infection indicated to be the susceptible population by comparing with controls. Besides, SNP rs3202538 (G/T) in ErbB3 3′-UTR was involved in the occurrence of GC by acting as tumor risk factors. SNP rs3202538 (G/T) could be regulated by both miR-204 and miR-211 which caused an upregulation of ErbB3 in patients. Furthermore, the carriers of T genotype was related to the significantly high expression of ErbB3, and to big tumor size, poor differentiation as well as the high probability of metastasis. Both miR-211 and miR-204 can significantly decrease cell proliferation, metastasis as well as downstream AKT activation through G but not T allele of ErbB3 3′UTR. Moreover, the SNP of G/T was associated with shorter survival of post-surgery GC patients with 5 years of follow up study.

**Conclusion:**

In conclusion, our findings have shown that the SNP rs3202538 (G/T) in ErbB3 3′-UTR acted as promotion factors in the GC development through disrupting the regulatory role of miR-204 and miR-211 in ErbB3 expression.

**Electronic supplementary material:**

The online version of this article (doi:10.1186/s12935-017-0449-z) contains supplementary material, which is available to authorized users.

## Introduction

Globally, gastric cancer (GC) is the fifth leading cause of cancer and the third leading cause of death from cancer making up 7% of cases and 9% of deaths. In 2012 GC occurred in 950,000 people and caused 723,000 deaths [1]. GC occurs most commonly in East Asia and Eastern Europe and it occurs twice as often in males as in females. The most common cause is infection by the bacterium *Helicobacter pylori* (*Hp*), which accounts for more than 60% of cases. The current diagnostic system was proved to be relatively poor in early-stage diagnosis GC, and accumulating evidence revealed the great potential of microRNAs (miRNAs) as biomarkers in GC diagnosis [2].

Receptor tyrosine-protein kinase ErbB-3, is encoded by the ErbB3 gene and a member of the epidermal growth factor receptor (EGFR/ERBB) family of receptor tyrosine kinases [3]. The kinase-impaired ErbB3 is known to form active heterodimers with other members of the ErbB family, most notably the ligand binding-impaired ErbB2 [4]. ErbB3 was reported to be overexpressed in human gastric cancer, it acts as a tumor promoter by activation of a serial of complicated signaling including PI3K/AKT, RAS/RAF/MAPK and etc. The diversity of expression of ErbB3 was related to its upstream regulation, among all this mechanism miRNA regulation was one of the important reasons. miR-125, miR-199a, miR-205, and miR-450 etc. were all reported to potentially capable of regulating ErbB3, generally by targeting its three prime untranslated regions (3′UTR) [5–9].

MicroRNAs (miRNAs) are a class of small, noncoding, approximately 22-nucleotides-long RNAs, which may function as a post-transcriptional regulator of gene expression. Single nucleotide polymorphism (SNP) is a variation in a single nucleotide that occurs at a specific position in the genome, where each variation is present to some appreciable degree within a population. It commonly occurs in the human genome, some SNP were functional associating with the structure of functional proteins. However, SNP also occurred in 3′UTR of certain genes which might effect on the binding force by various miRNA [10, 11].

Thus, in this study, we focused on the SNPs in the 3′UTR of ERBB3. By using the bioinformatics software (http://bioinfo.life.hust.edu.cn/miRNASNP/), we obtained all the SNPs which could regulate by miRNAs (Table 2). Via the bioinformatics prediction and statistical analysis, we found that the rs3202538 (G/T) in ErbB3 3′-UTR might potential effect on the regulation by miR-204 and miR-211. Moreover, a previous study has revealed that rs3202538 (G/T) in ErbB3 3′-UTR was seriously related to type I diabetes just via regulation of miR-204 and miR-211 [12], we further investigated the allele distribution in a case–control study.

## Materials and methods

### Study subjects

A total of 851 GC cases and sex plus age-matched 799 controls obtained from Zhenjiang Hospital of Chinese Traditional Medicine, were included in this study. Patients were consecutively recruited between February 2010 and January 2015. All cases are incident ones during enrollment of the current case–control study. The diagnosis of all patients was histological confirmed. A face-to-face questionnaire was administered to collect demographic data and environmental exposure information, including alcohol use and cigarette consumption status as well as family cancer history. The normal tissues sampled was obtained at least 2 cm away from the margin of the tumor. All participants have provided their written informed consents to participate in this study. This study was approved by the Institutional Review Board of Zhenjiang Hospital of Chinese Traditional Medicine.

### Genotype

The polymorphism was genotyped through the PCR-restriction fragment length polymorphism (RFLP) method as described previously [13]. The PCR reactions were carried out in a total volume of 5 μL containing TaqMan Universal Master Mix, 80X SNP Genotyping AssayMix, DNase-free water and 10 ng genomic DNA. The PCR conditions were 2 min at 50 °C, 10 min at 95 °C, followed by 40 cycles at 95 °C for 15 s and 60 °C for 1 min by the 384-well ABI 7900HT Real Time PCR System. A 10% random sample was reciprocally examined by different persons, and the reproducibility was 100%.

### Real-time PCR assay

Real-time polymerase chain reaction (RT-PCR) was performed to determine whether the mutation changed the expression level of ERBB3. The primers used for amplification were forward primer: CAGCAGCTTGACACACGGTA, and reverse primer: AAACACCAAAGTGGCATGTGA for ErbB3 and forward primer: TGTGGGCATCAATGGATTTGG, reverse primer: ACACCATGTATTCCGGGTCAAT for GAPDH. The amplification conditions were 95 °C for 10 min, followed by 40 cycles of 95 °C for 30 s, 55 °C for 40 s, and 72 °C for 30 s, and finally 4 °C for 30 min for cooling by the 384-well ABI 7900HT Real Time PCR System.

### Cell lines and cell culture

GC cell lines SGC-7901 and MKN-45 were purchased from the Chinese Academy of Sciences Cell Bank. All cells were cultured in Dulbecco’s Minimum Essential Medium (DMEM) purchased from Gibco (CA, USA) supplemented with 10% fetal bovine serum (Invitrogen, Carlsbad, USA) and grown in humidified 5% CO_2_ at 37 °C. MiR-214 and miR-1225a mimics and a normal control were obtained from Genepharma (Shanghai, China). The transfection was conducted by using Lipofectamine 2000 (Invitrogen Corp, CA, USA).

### Cell proliferation assays

Cell proliferation was determined by using CCK-8 (Dojin Laboratories, Kumamoto, Japan) according to the manufacturer’s instructions. Briefly, the control and infected cells were seeded at a density of 1 × 10^3^ cells/well in 96-well plates. 10 μL of CCK-8 was added to each well containing 100 µL of the culture medium, and the plate was incubated for 2 h at 37 °C. The viability of cells was evaluated by measuring the absorbance at 450 nm, using a microplate reader (Thermo Labsystems, CA).

### Western blot

For western blotting, proteins were extracted from tissues or cultured cells using RIPA buffer containing Protease Inhibitor Cocktails (P8340) (Sigma-Aldrich). An equal amount of proteins (100 μg) were separated with 7.5%/12.5% sodium dodecyl sulfate polyacrylamide gel electrophoresis (SDS-PAGE) and transferred to polyvinylidene fluoride (PVDF) membrane. Primary polyclonal antibodies targeting ErbB3 (ab20161), p-AKT (ab38449), AKT (ab8933), β-actin (ab3280) were purchased from Abcam (Cambridge, MA). The secondary antibodies were anti-rabbit or anti-mouse HRP-linked were purchased from Santa Cruz Biotechnology (CA, USA). The blots were developed using ECL reagent (Millipore, MASS, USA). An equal amount of protein loading in each lane was confirmed using β-actin antibody. ImageJ software quantified the integrated density of the band.

### Prediction of miRNAs binding to the SNP

Based on our bioinformatics analysis by using the bioinformatics software (http://www.bioguo.org/miRNASNP/) to predict the related SNPs in the 3′UTR of ERBB3 which could interact with miRNAs.

### Construction of luciferase-based reporter plasmids

A full-length fragment of the 3′UTR containing rs3202538 (G/T) (wild type/mutant) were amplified. The PCR product was cloned into the pGL3-promoter luciferase-based plasmid (Promega) at the cloning site between *Kpn*I and *Xho*I. The amplified fragment was verified by DNA sequencing. For cell proliferation and invasion assay, the full-length cDNA of ErbB3 was sub-cloned into the pGL3-ErbB3 3′UTR plasmid.

### Immunohistochemistry

Sections were de-paraffinized and followed by rehydration steps through a graded ethanol series and distilled water, and then were treated with 3% H2O2 in methanol for 30 min to block the endogenous peroxidase activity. The sections were rinsed in phosphate-buffered saline (PBS) twice, 5 min each time and incubated with 10% normal goat serum for 30 min to block non-specific antibody binding. After washing, the samples were incubated with primary anti-rabbit antibody ErbB3 (ab16901) purchased from Abcam (Cambridge, MA) at 4 °C overnight, and then washed with PBS for three times and then incubated with secondary antibodies. After that, the sections were stained with DAB according to manufacturer’s protocols and mounted and photographed using a digitalized microscope camera (Nikon, Tokyo, Japan).

### Dual-luciferase reporter assay

For luciferase activity analysis, SGC-7901 and MKN-45 cells were cotransfected with 100 ng of luciferase reporter constructs 5 ng of the β-gal control plasmid and 10 pmol of miRNAs with 1 µL Lipofectamine 2000 according to the manufacturer’s instructions (Invitrogen, NY, USA). After incubation for 48 h, we carried out the luciferase assay using the luciferase reporter assay system (Promega, Madison, WI) according to the manufacturer’s protocol. Measurements of luminescence and absorbance of β-gal were performed on a luminometer (Glomax 20/20; Promega). Three independent experiments were performed in triplicate.

### Cell invasion assay

For trans-well assay, the chamber was treated with Matrigel before cells were inoculated, 100 μL cell suspension with serum-free medium was seeded to the upper chamber, cells were stained with crystal violet staining solution (Beyotime, Nantong, China). Migrated cells were counted by using Image-pro Plus 6.0 while cell numbers of the normal control group were normalized to 1.

### Statistical analysis

Differences between cases and controls were evaluated by the Student’s t test for continuous variables and the χ2 test for categorical variables. The association between SNPs and GC risk was estimated by the OR and 95% CI using the general genetic model. The potential gene–environment interaction was evaluated by logistic regression analysis and tested by comparing changes in deviance between the models of main effects with or without the interaction term. The overall survival and relapse free survival rate in different groups were analyzed by using Kaplan–Meier curve. Comparisons between groups were analyzed by the t test (two-sided). All statistical analyses were performed using Prism Graphpad software.

## Results

### SNP rs3202538 in 3′UTR region of ErbB3 was a risk factor for development of gastric cancer

The differences in the distribution of the selected variables among GC and controls cases are listed in Table 1. No significant differences in age (P = 0.681) and sex (P = 0.286) were found between the case and the control group. There were more patients who had *Hp* infection in the GC patients than in the controls (P < 0.0001). However, all the variables above were further adjusted for any residual confounding effect in the later multivariate logistic regression analysis.

**Table 1:** Frequency distributions of selected variables in patients and cancer-free controls

Variables	Cases (*n* = 851)		Controls (*n* = 799)		P*
	*N*	%	*N*	%	
Age (years)					0.681
≤50	376	44.18	345	43.18	
>50	475	55.82	454	56.82	
Gender					0.286
Male	318	37.37	319	39.92	
Female	533	62.63	480	60.08	
Hp infection					<0.0001
Negative	254	29.85	550	68.84	
Positive	597	70.15	249	31.16	
Differentiation grade					
Poor	214	25.15			
Moderate	294	34.55			
Well	343	40.31			
Tumor size (cm)					
≤3	276	32.43			
>3	575	67.57			
Metastasis					
Yes	412	48.41			
No	439	51.59			

* Two-sided Chi square test for either genotype distributions or allele frequencies between cases and controls

To investigate the miRNA associated SNPs in the 3′UTR of ERBB3, we first found all the possible SNPs from the SNP databases NCBI db SNP BUILED 129 and ENSEMBL v58 in the 3′-UTR of ErbB3 gene with the minor allele frequency (MAF) >0.05. And then used bioinformatics software Diana-Micro and RNA hybrid to predict miRNAs that can bind to the ErbB3 3′-UTR (data not shown). Finally, we obtained five SNPs in 3′UTR of ErbB3 which could be regulated by different miRNAs (Table 2). The positions of the SNPs in 3′UTR of ERBB3 as well as the variants were also listed. Further genotyping was performed to detect the distribution of allele gene of the SNP in our research, among all this SNPs, rs3202538 which can be potentially regulated by miR-204 miR-211 and miR-4278 were in Hardy–Weinberg equilibrium distribution pattern in the healthy control group (P < 0.0001).

**Table 2:** List of SNP in 3′UTR region of ErbB3

SNP	chr	3′UTR position	Associated miRNA	Allele
rs3202538	12	373–395	hsa-miR-204	G/T
rs3202538	12	373–395	hsa-miR-211	G/T
rs3202538	12	373–395	hsa-miR-4287	G/T
rs78743019	12	1076–1101	hsa-miR-3170	A/C
rs35291818	12	102–119	hsa-miR-4314	–/C

Logistic regression analyses indicated that individuals with the GT and TT of rs3202538 in 3′UTR of ErbB3 were significantly associated with GC risk (P < 0.0001), which indicated that this SNPs might be a risk factor in GC development. Moreover, the significant association with GC risk was shown in U carrier of both SNPs (P < 0.0001) (Table 3). For SNP rs3202538, besides Wild type GG, other SNPs in 3′UTR of ErbB3 were significantly associated GC risk, especially for TT genotype (for TT: OR 4.32, 95% CI 1.34–1.88; for GT: OR 1.89, 95% CI 1.48–2.01; and for G carrier: OR 3.75; 95% CI 1.47–2.04). All ORs were adjusted for sex, age, and *Hp.* Infection status, or family cancer history.

**Table 3:** Genotype frequencies of the ErbB3 at rs3202538 polymorphism among GC cases and controls

Genotype	Cases (*n* = 851)		Controls (*n* = 799)		OR (95% CI)^a^	*P* value^a^
	*N*	%	*N*	%		
rs3202538						
GG	178	20.92	398	49.81	1	<0.0001
GT	317	37.25	217	27.16	1.89 (1.48–2.01)	
TT	356	41.83	184	23.03	4.32 (1.34–1.88)	
T carrier	673	79.08	401	50.19	3.75 (1.47–2.04)	<0.0001

^a^The ORs, 95% CIs and *P* value were calculated after adjusting for age, gender, parental Hp infection history and family cancer history

### SNP rs3202538 in 3′UTR region of ErbB3 was associated with GC clinical characters by deregulation of miR-204 and miR-211

Then, we did a stratified analysis of the association of the rs3202538 with the clinic pathological parameters of GC (Table 4). We found a significant association of the both genotypes with the tumor size, differentiation and metastasis as well as. Compared with the wild type, the carriers of T genotype presented significant large tumor size, poor differentiation as well as the high potential of metastasis (Table 4).

**Table 4:** Stratified analysis of genotypes of ErbB3 rs3202538 with clinicopathological parameters of GC

Features	Genotype			GG vs GT *P* value*	GG vs TT *P* value*
	GG	GT	TT		
Age (years)				0.167	0.081
≤50	81	161	134		
>50	97	156	222		
Gender				0.416	0.756
Male	75	137	145		
Female	103	219	211		
*Hp.* infection				0.002	0.169
Negative	48	129	77		
Positive	130	188	279		
Differentiation grade				0.0047	<0.0001
Well	73	94	47		
Moderate	68	117	120		
Poor	37	106	189		
Tumor size (cm)				0.109	<0.0001
≤3	93	164	19		
>3	85	153	337		
Metastasis				0.0042	<0.0001
Yes	49	128	235		
No	129	189	121		

* Two-sided Chi square test for either genotype distributions or allele frequencies between cases and controls

**Fig. 1:**
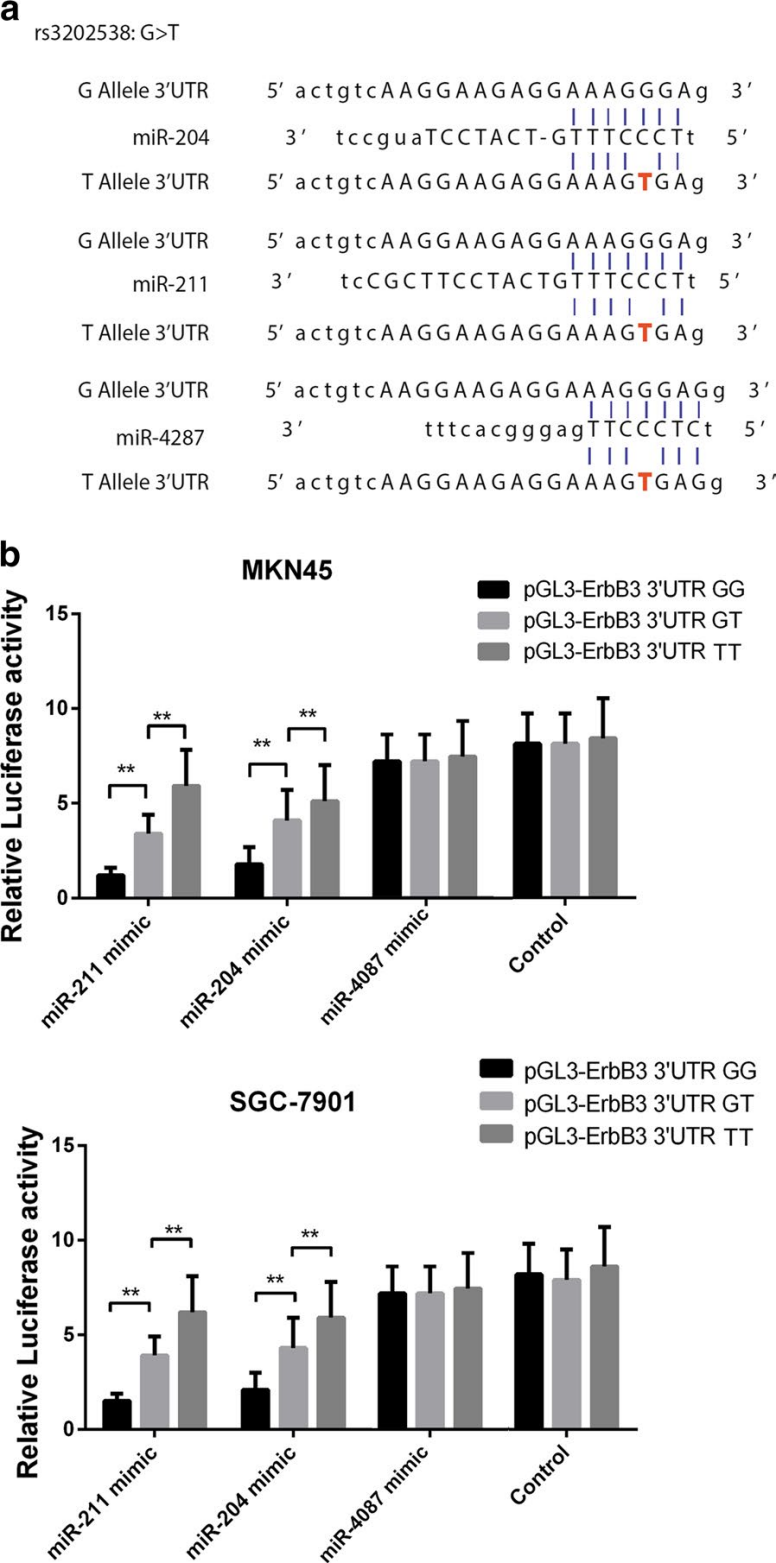
SNP rs3202538 in ERBB3 3′UTR of GC patients with GT/TT genotype can up-regulate of ERBB3 transcription by deregulated by miR-211 and miR-204. **a** Bioinformatics predicted the binding site of the miR-204, miR-211 and miR-4287 with ErbB3 and the mutation types were conducted into the pGL3 plasmid as presented. **b** Cells were cotransfected with miR-204, miR-211 and miR-4287 mimics or control, Renilla luciferase vector pRL-SV40 for 48 h. Both firefly and Renilla luciferase activities were measured in the same sample. Firefly luciferase signals were normalized with Renilla luciferase signals. Data were presented as the mean ± SEM. Asterisk indicates a significant difference (P < 0.05)

To investigate whether the rs3202538 SNPs affects the predicted miRNAs combining with ErbB3 3′UTR (Fig. 1a), we performed transient transfection in vitro and measured the relative activities with a Dual-Glo Luciferase Reporter Assay System. As it was indicated, co-transfection of the luciferase vector containing either wild type (GG) and homozygous T mutant 3′UTR of ErbB3 plus miR-204, miR-211 and miR4287 mimics and control into two GC cells, significantly increase the luciferase expression level was found in the mutant compared to wild types in both miR-204 and miR-211 but not miR-4087 (Fig. 1b). So it is indicated that the SNP in 3′UTR of ERBB3 can affect the binding affinity of either miR-204 and miR-211, and then affect the function of post-transcriptional regulation, resulting in the abnormal expression level of ErbB3.

### G>T of rs3202538 promote cell proliferation and invasion by enhanced ErbB3 expression in miR-211 or miR-204 overexpression GC cell

**Fig. 2:**
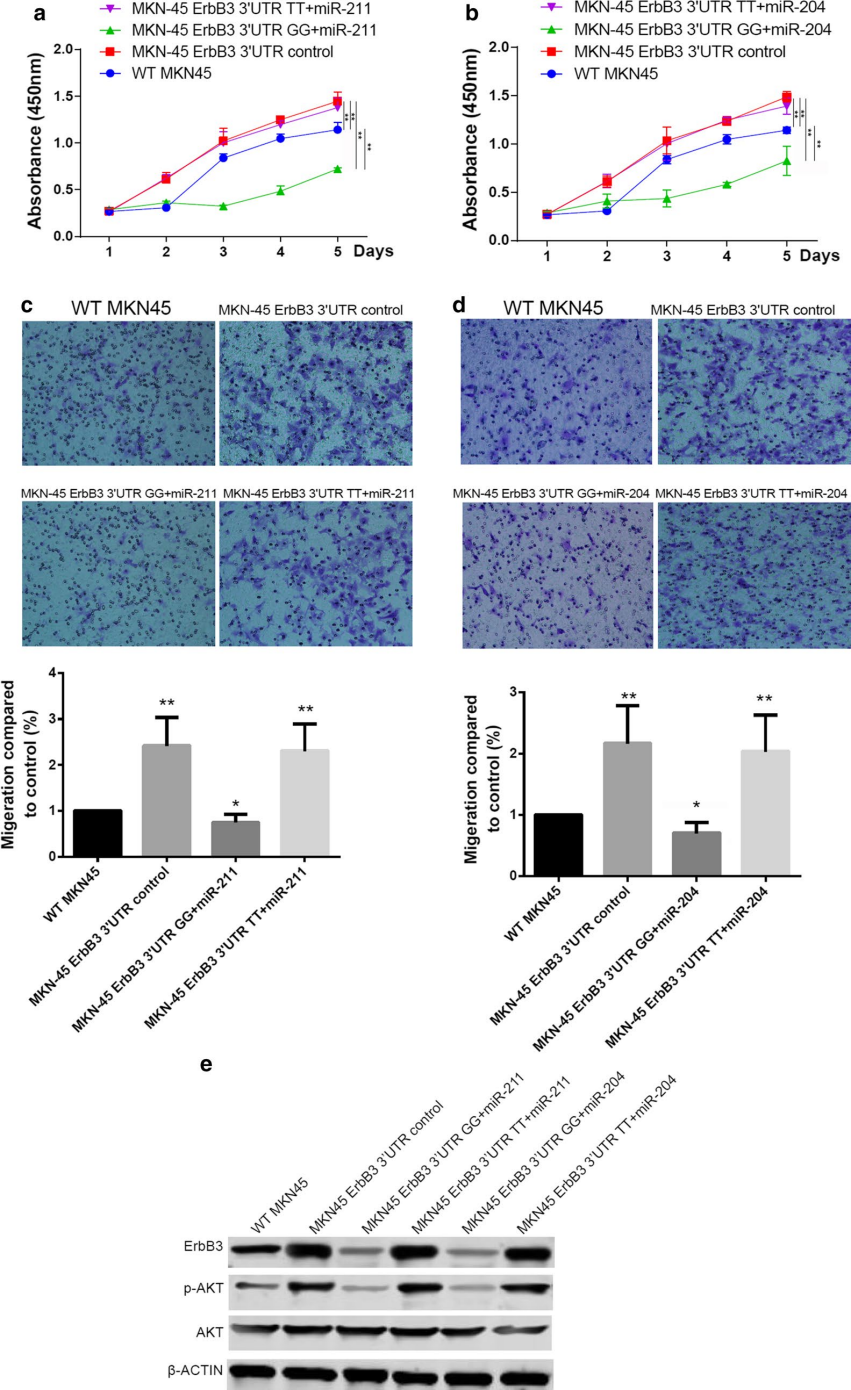
G>T of rs3202538 promote cell proliferation and invasion by enhanced ErbB3 expression in miR-211 or miR-204 overexpression GC cell. **a**, **b** The cell proliferation was determined for 5 days of the cells treated differently indicated in the figures. **c**, **d** The cell metastasis capability of variously treated MKN45 cells were determined by using a trans-well based cell invasion assay. **e** The expression of ErbB3 as well as activation of AKT signaling pathway in cells treated differently were determined by using western-blot. Data were presented as the mean ± SEM. *P < 0.05 and **P < 0.01

To investigate the effect of G>T SNP on cell proliferation and metastasis, we overexpressed ErbB3 in MKN45 cells using the pGL3 vector, overexpression of ErbB3 can significantly increase the cell proliferation and cell invasion compared to the WT control. Both miR-211 and miR-204 can significantly decrease the cell proliferation and invasion ability in the G allele 3′UTR even lower than the WT control. However, there is no significant difference in both cell proliferation and invasion between the ErbB3 overexpressed MKN45 and MKN45 overexpressed T allele 3′UTR regulated ErbB3. This result indicated that the post-transcriptional regulation by miR-204 and miR-211 can be attenuated dramatically due to the G>T SNP in 3′UTR of ErbB3 (Fig. 1a–d). We also detected the expression of ErbB3 as well as its classical downstream AKT signaling by using western-blot, the protein expression of ErbB3 and the phosphorylated AKT in the residue of T308 can be significantly decreased in the G allele 3′UTR regulated ErbB3 overexpression MKN cells regulated by both miR-204 and miR-211. However, miR-204 and miR-211 have no apparent effects on the expression of ErbB3 or phosphorylated T308 AKT was investigated in the MKN45 cell overexpressed T allele 3′UTR regulated ErbB3 (Fig. 2e).

### SNP rs3202538 in 3′UTR region of ErbB3 was contributed to upregulation of human GC ErbB3 and associated with poor prognosis of post-surgery GC patients

**Fig. 3:**
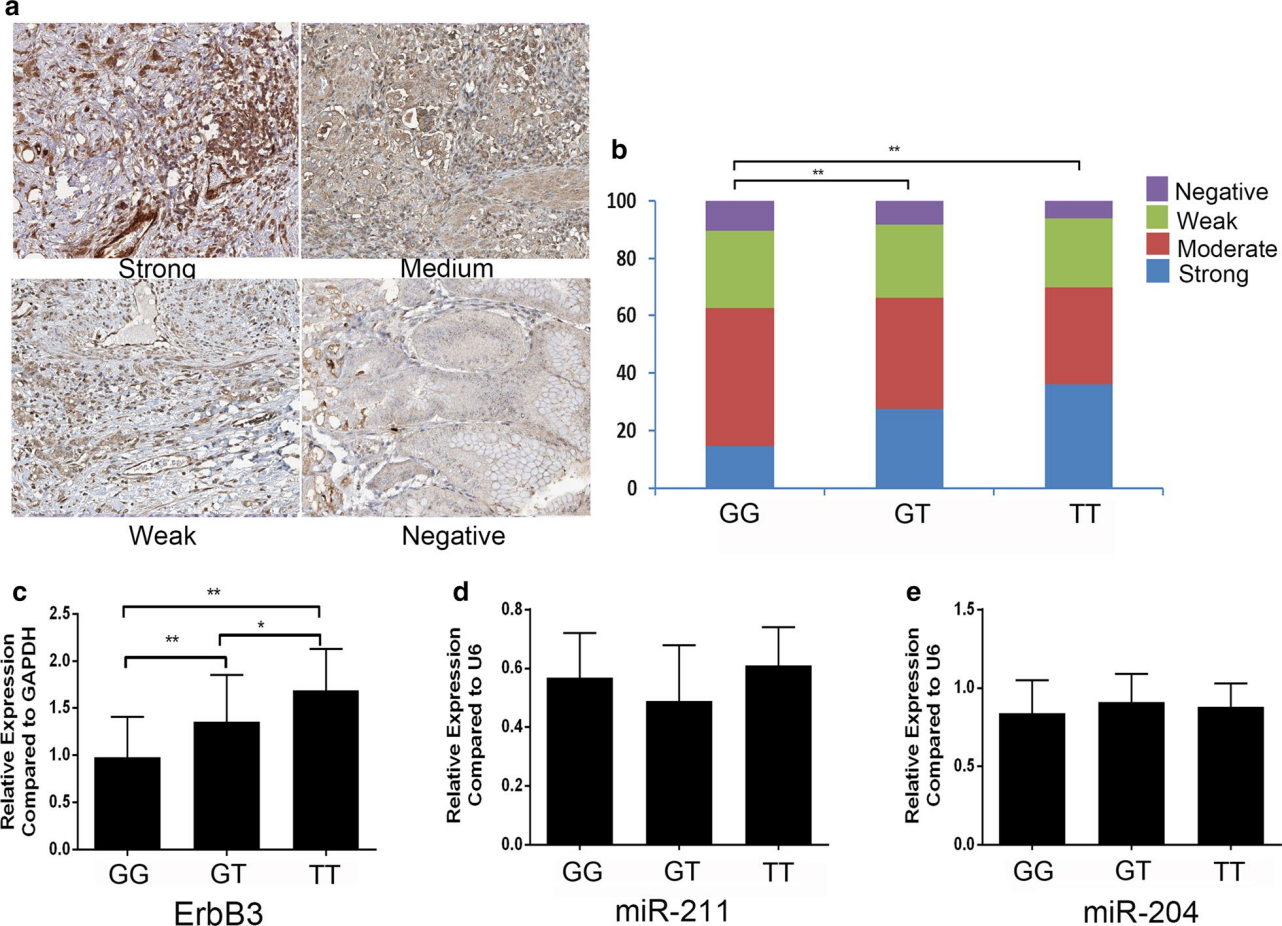
SNP rs3202538 in ERBB3 3′UTR on the expression of ERBB3 in clinical samples. **a** Representative figures for IHC staining of ERBB3 in GC tumor section. **b** Comparison of a component of IHC staining in both in TT, GT and GG genotype GC patents. **c**, **d**, **e** The expression level of ERBB3, miR-211 and miR-204 were determined by real-time PCR in different genotypes GC patents

We also confirmed the expression of ERBB3 in clinical samples with different genotypes of rs3202538. ErbB3 expression was detected in human GC by IHC, there are strong, medium, weak and negative staining in varying sections, GG group ERBB3 staining components were significantly different to that in GT and TT group in ErbB3 expression (high 14.6%, medium 48.2% weak 26.8% and negative 10.4% for GG group; and high 27.6%, medium 38.5%, weak 25.7% and negative 8.2% for GT; high 36.1%, medium 33.6% weak 24.1% and negative 6.2% for GG group, P < 0.001) (Fig. 3a, b). Real-time PCR further confirmed such difference in ERBB3 transcription, however, there is no significant difference in miR-204 and miR-211 expression between three groups (Fig. 3c–e).

**Fig. 4:**
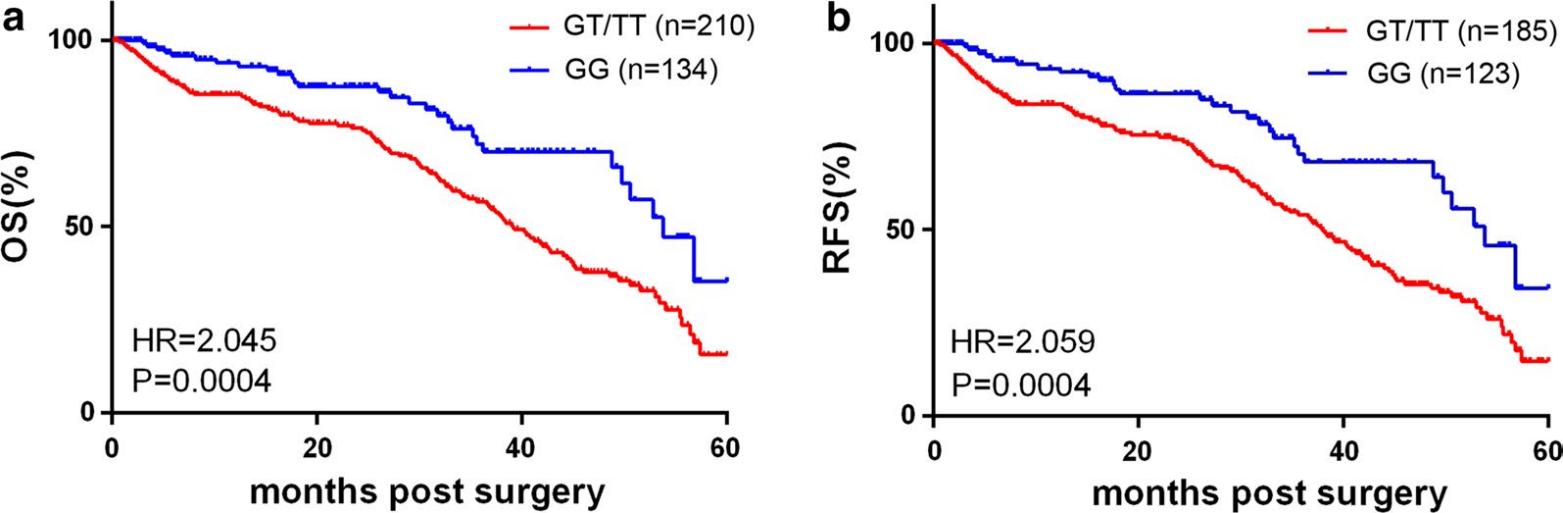
SNP rs3202538 in ERBB3 3′UTR on overall survival and relapse free survival. **a**, **b** Overall survival rate and relapse free survival of post-surgery GC patients were analyzed by Kaplan–Meier survival curve. Data were presented as the mean ± SEM. *P < 0.05 and **P < 0.01

Among of total 851 GC patients we have 344 patients with follow up data of survival and 36 GC patients have found reoccurrence. Therefore, survival analysis was performed in GG and U carrier group. Firstly, the overall 5-year survival rate in the TT/GT group was lowest as 15.72% which significantly different to GG group with a higher survival rate of 35.38% (P = 0.0004, HR = 2.045, 95% CI 1.321–2.644) (Fig. 4a, Additional file 1). The relapse free survival (RFS) was also investigated by Kaplan–Meier curve, and it is similar to the OS analysis that RFS in GG is also significantly higher than the T carrier group (P = 0.0004, HR = 2.059, 95% CI 1.329–2.659) (Fig. 4b, Additional file 2). In general, our foundlings indicated that the G/T SNP might serve as a tumor promoter and poor prognosis indicator in GC by affecting the binding of miR-204 and miR-211 on the 3′UTR of ErbB3.

## Discussion

Overexpression of ErbB3 was reported in human gastric cancer in several independent types of research, and also a high expression of ErbB3 was regarded as an indicator of poor prognosis in post-surgery patients [3, 4, 14]. Recent works have implied the role of ErbB3 in gastric cancer as a key signaling hub [15]. On the one hand, HER3 overexpression may promote tumor progression and invasion by activation of PI3K/AKT signaling pathway [16]. On the other hand, a lot of direct evidence has emerged the benefit of anti-HER3 agents in combination with EGFR tyrosine kinase inhibitors as well as anti-HER2 agents in gastric cancer [17]. And moreover, alternative activation of c-MET mediated signaling was also dependent on ErbB3, which reflected as the development of resistance to c-MET inhibitors may result from the overexpression of ErbB3 [18].

The expression of ErbB3 was diverse in different GC patients, therefore, the signaling activation was also different. So far there was rarely research addressing the diversity of expression of ErbB3 in human GC, we addressed this issue here as SNP in its 3′UTR region and might cause deregulation of certain miRNA. miR-204 and miR-211 were found to have a potential suppression effect on ErbB3 transcription and SNP rs3202538 from G to T might increase ErbB3 expression by actuating such suppressive effect. In the present study, the difference of ErbB3 expression was verified between the G and T allele, the expression of ErbB3 can be attenuated dramatically by both miR-204 and miR-211 even in exogenous ErbB3 overexpression cells regulated by G allele but not the T allele 3′UTR. Also, the cell proliferation, invasion ability as well as downstream pathway activation also decreased corresponding to the expression of ErbB3.

The roles of miR-211 were complicated and controversial. Some of the researchers implied it as a tumor suppressor by targeting oncogenic genes such as IGF2R, TGFBR2 and NFAT5 in melanoma [19]. And moreover, in human breast cancer, miRNA-211 directly inhibit CDC25B expression in breast cancer cells, alters other related target proteins CCNB1 and FOXM1, and then inhibits breast cancer cells growth, migration, and invasion and lead G2/M arrest [20]. However, some studies defined miR-211 as a tumor promoter, by inhibiting the expression of SRC kinase signaling inhibitor 1 (SRCIN1), miR-211 was proved to promote human non-small cell lung cancer [21], and by targeting tumor suppressive gene CHD 5, miR-211 can promote human colon cancer [22, 23]. However, our study indicated that mIR-211 as a tumor suppressor in human GC by targeting ErbB3.

Compared to miR-211, the role of miR-204 was more explicit. In 2014 Zhou et al. reported that *Hp* infection can down-regulate expression of miR-204 and thus to promote human GC by targeting SOX4 [24], And miR-204 was extensively reported to suppression of EMT by targeting varying genes including snail and sirt1 [25, 26]. And in the present study, we revealed that miR-204 can also relate to metastasis of human GC by targeting ErbB3.

## Conclusion

In the present study, we found miR-211 and miR-204 have a potential transcriptional suppressive effect on ErbB3, and SNP in 3′UTR region of ErbB3 can effectively associate significantly up-regulation of ErbB3 which might occur due to loss regulation of miRNA. These findings are novel and might contribute to explicit the individual differences in GC susceptibility.

## Additional files

**Additional file 1.** The output files of overal survival rate comparison in different groups analyzed by Kaplan–Meier survival curve.

**Additional file 2.** The output files of relapse free survival rate comparison in different groups analyzed by Kaplan–Meier survival curve.

## Abbreviations

GC: gastric cancer
SNP: single-nucleotide polymorphism
3′UTR: three prime untranslated region
95% CI: 95 confidence interval
HR: hazard ratio
OD: odds ratio
CC: CC genotype
AA: AA genotype
C/A: SNP C to A
